# Supplementary material for: Identification of photoperiod-induced specific miRNAs in the adrenal glands of Sunite sheep (Ovis aries)
Source: Front Vet Sci. 2022 Jul 22;9:888207. doi: 10.3389/fvets.2022.888207 (PMC9354845; doi:10.3389/fvets.2022.888207)
Supplement: Supplementary file 1 [file Data_Sheet_1.ZIP › Supplementary file/miRDeep2 file.zip!/miRDeep2 file/Novel_Structure/SPLP42A1/Novel_274.pdf]

Provisional ID : NC_019467.2_15785
Score total : 37.3
Score for star read(s) : 3.9
Score for read counts : 29.7
Score for mfe : 2.1
Score for randfold : 1.6
Score for cons. seed :
Total read count : 70
Mature read count : 69
Loop read count : 0
Star read count : 1

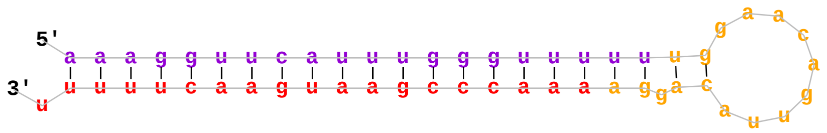

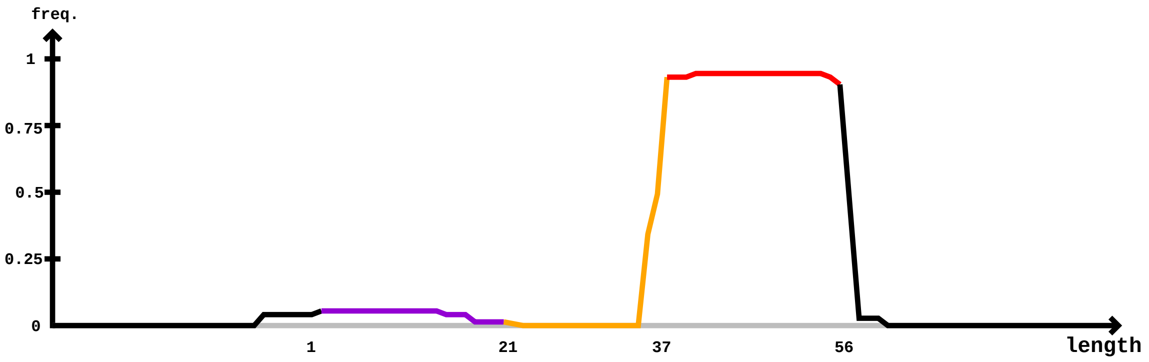

Star Mature

```
5'- ccuguuuccauguauuggguuggcugaaagguucauuuggguuuuuuggaacaguuacaggaaaacccgaaugaacuuuuuggccuacccaguaagucucuguucaucc  -3'  obs
    ccuguuuccauguauuggguuggcugaaagguucauuuggguuuuuuggaacaguuacaggaaaacccgaaugaacuuuuuggccuacccaguaagucucuguucaucc        exp
    ............((((((((.(((((((((((((((((((((((((((.........)).))))))))))))))))))))))))).))))))))...............  reads  mm  sample
    ....................uggcugaaagguucauuug......................................................................  1  0  seq
    ....................uggcugaaaAguucauuugggu...................................................................  2  1  seq
    ..........................aaagguucauuuggguuuuu...............................................................  1  0  seq
    ............................................................gaaaaccUgaaugaacuuuu.............................  1  1  seq
    ............................................................gaaaaccUgaaugaacuuuuu............................  1  1  seq
    ............................................................gaaaaccAgaaugaacuuuuug...........................  1  1  seq
    ............................................................gaaaacccAaaugaacuuuuug...........................  18  1  seq
    ............................................................gaaaacccgaaugaacuuuuuA...........................  3  1  seq
    ............................................................gaaaaccUgaaugaacuuuuug...........................  1  1  seq
    .............................................................aaaaccAgaaugaacuuu..............................  1  1  seq
    .............................................................aaaaccAgaaugaacuuuu.............................  1  1  seq
    .............................................................aaaaccGgaaugaacuuuuu............................  1  1  seq
    .............................................................aaaaccAgaaugaacuuuuu............................  3  1  seq
    .............................................................aaaacccgaaugaacuuuuug...........................  5  0  seq
    ..............................................................aaacccgaaugaacuuuuC............................  1  1  seq
    ..............................................................aaacccgaaugaacuuuuu............................  26  0  seq
    ..............................................................aaacccgaaugaacuuuuCg...........................  2  1  seq
    ..............................................................aaaccGgaaugaacuuuuug...........................  1  1  seq
    ..............................................................aaacccgaaugaaUuuuuug...........................  1  1  seq
    ..............................................................aaacccgaaugaacuuuuuggcA........................  1  1  seq
    .................................................................cccgaaugaacuuuuuggcA........................  1  1  seq
```
